# Supplementary material for: SARS-CoV-2 Virus-Like Particle Neutralizing Capacity in Blood Donors Depends on Serological Profile and Donor-Declared SARS-CoV-2 Vaccination History
Source: Microbiol Spectr. 2022 Feb 16;10(1):e02262-21. doi: 10.1128/spectrum.02262-21 (PMC8849073; doi:10.1128/spectrum.02262-21)
Supplement: SUPPLEMENTAL FILE 1 — Supplemental material. Download SPECTRUM02262-21_Supp_1_seq8.pdf, PDF file, 0.2 MB [file spectrum02262-21_supp_1_seq8.pdf]

SUPPLEMENTAL DATA

TABLE S1. Summary of VLP neutralization data expressed as $\log_2(ID_{50})$ classified into vaccinated and unvaccinated donor groups (January-March 2021).

Classification	Specimen #	Wild type	Alpha	Beta	Gamma	Delta
Vaccinated and anti-N	CIHR013654	7.24	5.07	0	0	3.44
	CIHR015946	5.31	0	0	0	0
	CIHR016894	15.81	14.64	13.55	14.70	14.88
	CIHR017333	12.12	11.21	8.13	10.73	11.54
	CIHR017730	13.98	12.83	11.96	12.39	12.61
Vaccinated and no anti-N	CIHR013818	11.06	10.59	0	0	11.37
	CIHR014329	7.94	6.80	4.78	6.52	7.80
	CIHR015234	6.75	0	0	0	0
	CIHR015533	14.16	12.65	9.41	11.63	12.33
	CIHR015657	8.63	6.79	5.28	6.96	7.23
	CIHR015884	10.95	9.48	7.70	9.96	10.32
	CIHR015958	10.13	7.53	0	6.30	7.57
	CIHR016698	10.53	9.98	6.94	9.61	9.46
	CIHR016904	6.35	4.44	0	0	5.75
	CIHR016905	12.20	11.24	8.96	9.95	11.22
	CIHR016930	0	0	0	0	0
	CIHR017087	8.49	6.90	6.27	8.29	8.66
	CIHR017189	10.21	8.73	6.52	9.30	9.00
	CIHR017229	0	0	0	0	0
	CIHR017534	5.79	5.61	0	0	0
	CIHR017540	9.39	7.14	0.000	6.67	6.22
	CIHR017728	13.47	12.21	9.71	11.21	11.38
	CIHR017824	10.45	8.75	8.05	9.35	8.27
	CIHR017838	13.19	11.45	9.28	10.83	10.98
	CIHR018126	9.64	9.13	6.85	9.01	8.27
Unvaccinated and anti-N	CIHR013757	0	0	0	0	0

	CIHR013936	8.94	7.60	6.34	7.17	7.28
	CIHR014110	9.46	6.97	7.84	8.62	6.96
	CIHR014113	10.31	7.02	5.66	8.24	7.14
	CIHR014235	9.98	8.55	5.22	5.96	9.90
	CIHR014309	8.40	7.01	5.66	7.55	7.02
	CIHR014834	6.05	5.37	0	0	5.29
	CIHR014840	7.53	6.57	4.54	5.45	5.76
	CIHR014884	9.14	6.92	4.96	8.36	6.93
	CIHR014993	0	0	0	0	0
	CIHR015094	11.02	10.13	11.82	10.52	12.11
	CIHR015434	8.08	6.78	5.66	7.25	7.15
	CIHR016024	8.07	8.41	0	5.17	8.73
	CIHR016624	6.19	5.71	4.68	5.53	6.39
	CIHR016979	8.13	7.81	0	8.85	10.12
	CIHR017127	6.19	5.13	0	2.97	0.00
	CIHR017305	8.00	7.14	5.22	7.20	7.17
	CIHR017724	11.33	10.29	8.52	9.57	9.12
	CIHR017894	6.46	6.33	5.29	5.80	5.40
	CIHR017990	8.55	7.19	5.36	6.88	6.01
Unvaccinated and no anti-N	CIHR014238	0	0	0	0	0
	CIHR014491	0	0	0	0	0
	CIHR014632	0	0	0	0	0
	CIHR014664	0	0	0	0	0
	CIHR014926	0	0	0	0	0
	CIHR015079	0	0	0	0	0
	CIHR015475	0	0	0	0	0
	CIHR015843	0	0	0	0	0
	CIHR015948	0	0	0	0	0
	CIHR016403	0	0	0	0	0
	CIHR016447	0	0	0	0	0
	CIHR016548	0	0	0	0	0

	CIHR016557	0	0	0	0	0
	CIHR016973	0	0	0	0	0
	CIHR017530	0	0	0	0	0
	CIHR017945	0	0	0	0	0
	CIHR018000	0	0	0	0	0
	CIHR018002	0	0	0	0	0
	CIHR018166	0	0	0	0	0
	CIHR018178	0	0	0	0	0

TABLE S2 A descriptive summary of transformed VLP neutralization data ($\log_2[ID_{50}]$) based on donor vaccine history and plasma anti-N positivity

Category		Wild type	Alpha	Beta	Gamma	Delta
Vaccinated and anti-N	Number of values	5	5	5	5	5
	Minimum $\log_2(ID_{50})$	5.3	0	0	0	0
	25% Percentile $\log_2(ID_{50})$	6.3	2.5	0	0	1.7
	Median $\log_2(ID_{50})$	12.1	11.2	8.1	10.7	11.5
	75% Percentile $\log_2(ID_{50})$	14.9	13.7	12.8	13.5	13.7
	Maximum $\log_2(ID_{50})$	15.8	14.6	13.6	14.7	14.9
	Range $\log_2(ID_{50})$	10.5	14.6	13.6	14.7	14.9
Vaccinated and no anti-N	Number of values	20	20	20	20	20
	Minimum $\log_2(ID_{50})$	0	0	0	0	0
	25% Percentile $\log_2(ID_{50})$	7.0	5.9	0	0	5.87
	Median $\log_2(ID_{50})$	9.9	8.1	5.8	7.6	8.3
	75% Percentile $\log_2(ID_{50})$	11.0	10.4	8.0	9.9	10.8
	Maximum $\log_2(ID_{50})$	14.2	12.7	9.7	11.6	12.3
	Range $\log_2(ID_{50})$	14.2	12.7	9.7	11.6	12.3
Unvaccinated and anti-N	Number of values	20	20	20	20	20
	Minimum $\log_2(ID_{50})$	0	0	0	0	0
	25% Percentile $\log_2(ID_{50})$	6.3	5.9	0	5.2	5.5
	Median $\log_2(ID_{50})$	8.1	7.0	5.2	7.0	7.0
	75% Percentile $\log_2(ID_{50})$	9.4	7.8	5.7	8.3	8.4
	Maximum $\log_2(ID_{50})$	11.3	10.3	11.8	10.5	12.1
	Range $\log_2(ID_{50})$	11.3	10.3	11.8	10.5	12.1
Unvaccinated and no anti-N	Number of values	20	20	20	20	20
	Minimum $\log_2(ID_{50})$	0	0	0	0	0
	25% Percentile $\log_2(ID_{50})$	0	0	0	0	0
	Median $\log_2(ID_{50})$	0	0	0	0	0
	75% Percentile $\log_2(ID_{50})$	0	0	0	0	0

	Maximum $\log_2(ID_{50})$	0	0	0	0	0
	Range $\log_2(ID_{50})$	0	0	0	0	0

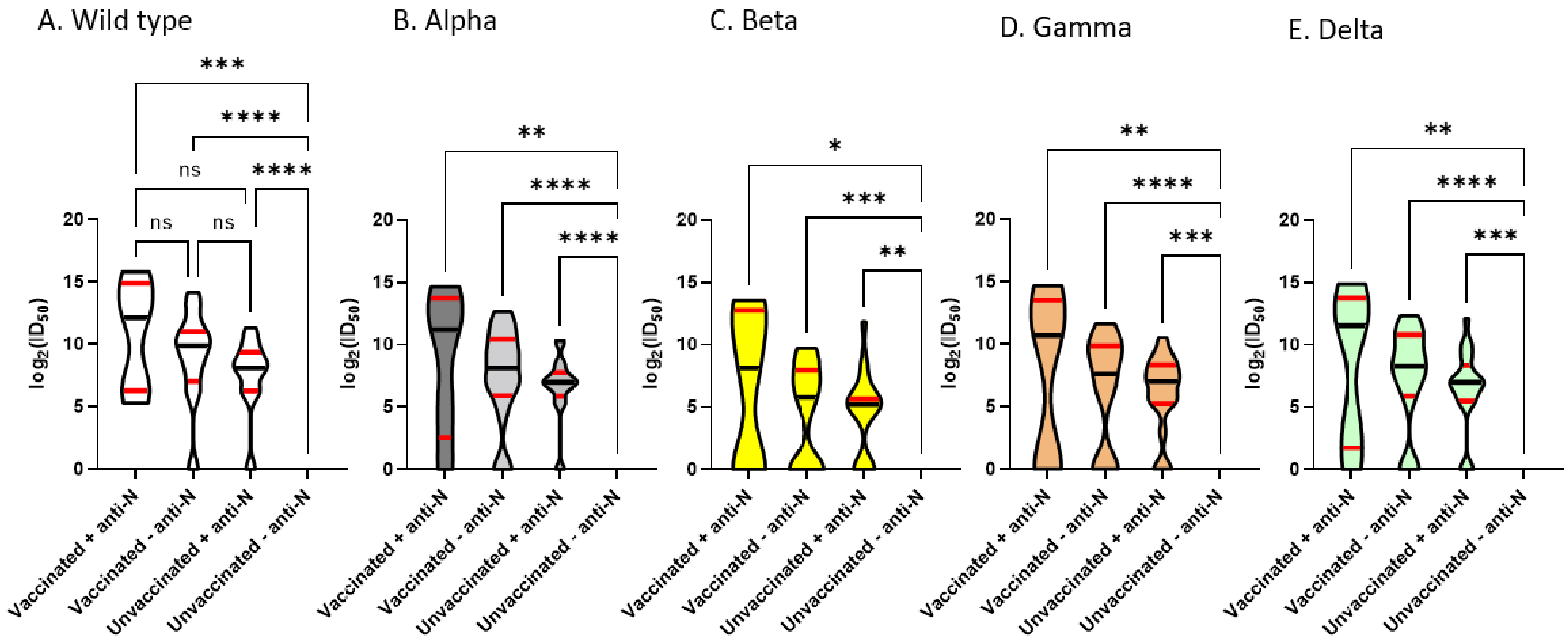

FIG S1

FIG S1 Differences in neutralization of Wild type, Alpha, Beta, Gamma and Delta VLPs between vaccinated and unvaccinated groups. Data are represented as violin plots with medians (black horizontal lines), 25-percentiles (lower red horizontal lines) and 75-

percentiles (upper red horizontal lines). Truncated violin plots range from minimum to maximum data points. For panel A) Wild type and all VOC VLPs (Alpha, [panel B], Beta, [panel C], Gamma [panel D], and Delta [panel E]) a significant reduction in “unvaccinated and no anti-N” was noted when compared to “vaccinated and anti-N,” “vaccinated and no anti-N”, and “unvaccinated and anti-N” groups. *Represents significance difference in neutralizing capacity of plasma against wild type and variant VLPs as determined by Dunn’s multiple comparisons.

Differences in neutralization of Wild type, Alpha, Beta, Gamma and Delta VLPs between vaccinated and unvaccinated groups

Differences in neutralization of Wild type, Alpha, Beta, Gamma and Delta VLPs between vaccinated and unvaccinated groups were analyzed using transformed VLP neutralization ($log_2[ID_{50}]$) from Table S1. A descriptive summary of the $log_2(ID_{50})$ transformed neutralization data is listed in Table S2. In Fig. S1A-E, truncated violin plots range from minimum to maximum data points described in Table S2.

For Wild type VLP neutralization (Fig. S1), there was a significant difference between vaccinated and unvaccinated groups (Kruskal-Wallis test statistic 38, $p<0.0001$, approximate). Neutralization of Wild-type VLPs was significantly reduced for “unvaccinated and no anti-N” when compared to; “vaccinated and anti-N” (Dunn’s multiple comparison test, $p=0.0004$), “vaccinated and no anti-N” (Dunn’s multiple comparison test, $p<0.0001$), and “unvaccinated and anti-N” (Dunn’s multiple comparison test, $p<0.0001$) (Fig. S1A).

For Alpha VLP neutralization (Fig. S1B), there was a significant difference between vaccinated and unvaccinated groups (Kruskal-Wallis test statistic 34, $p<0.0001$, approximate). Neutralization of Wild-type VLPs was significantly reduced for “unvaccinated and no

anti-N” than for; “vaccinated and anti-N” (Dunn’s multiple comparison test, p=0.0024), “vaccinated and no anti-N” (Dunn’s multiple comparison test, p<0.0001), and “unvaccinated and anti-N” Dunn’s multiple comparison test, p<0.0001) (Fig. S1B).

For Beta VLP neutralization (Fig. S1C), there was a significant difference between vaccinated and unvaccinated groups (Kruskal-Wallis test statistic 21, p=0.0001, approximate). Neutralization of Beta VLPs was significantly reduced for “unvaccinated and no anti-N” than for; “vaccinated and anti-N” (Dunn’s multiple comparison test, p=0.015), “vaccinated and no anti-N” (Dunn’s multiple comparison test, p=0.0008), and “unvaccinated and anti-N” Dunn’s multiple comparison test, p<0.0017) (Fig. S1C).

For Gamma VLP neutralization (Fig. S1D), there was a significant difference between vaccinated and unvaccinated groups (Kruskal-Wallis test statistic 27, p<0.0001, approximate). Neutralization of Beta VLPs was significantly reduced for “unvaccinated and no anti-N” than for; “vaccinated and anti-N” (Dunn’s multiple comparison test, p=0.0095), “vaccinated and no anti-N” (Dunn’s multiple comparison test, p<0.0001), and “unvaccinated and anti-N” Dunn’s multiple comparison test, p<0.0002) (Fig. S1D).

For Delta VLP neutralization (Fig. S1E), there was a significant difference between vaccinated and unvaccinated groups (Kruskal-Wallis test statistic 31, p<0.0001, approximate). Neutralization of Beta VLPs was significantly reduced for “unvaccinated and no anti-N” than for; “vaccinated and anti-N” (Dunn’s multiple comparison test, p=0.0023), “vaccinated and no anti-N” (Dunn’s multiple comparison test, p<0.0001), and “unvaccinated and anti-N” Dunn’s multiple comparison test, p<0.0002) (Fig. S1E).
